# Protocols for Traditional Chinese Medicine guidelines for acute primary headache

**DOI:** 10.2478/jtim-2023-0082

**Published:** 2023-07-05

**Authors:** Yuning Yao, Shuqin Liao, Zhengguo Cheng, Kegang Cao

**Affiliations:** Dongzhimen Hospital, Beijing University of Chinese Medicine, Beijing 100700, China; Institute for Brain Disorders, Beijing University of Chinese Medicine, Beijing 100700, China

**Keywords:** acute primary headache, Traditional Chinese Medicine, treatment, guidance, protocol

## Abstract

To further improve clinical workers’ Traditional Chinese Medicine (TCM) treatment level for headache attack, the TCM Guidelines for Acute Primary Headache has been developed based on the development methodology of the World Health Organization Standard Version guide. The grading of recommendations assessment, development and evaluation (GRADE) method was adopted for the development of evidence, evidence classification, and recommendations that can be systematically evaluated. For evidence lacking clinical research support, the quality of evidence was evaluated and recommended based on the evidence level standard of ancient books of traditional Chinese medicine, and The Appraisal of Guidelines for Research and Evaluation II (AGREE II) and The Reporting Items for Practice Guidelines in Healthcare (RIGHT) were referred to. This guideline plan mainly introduces the guideline formulation process of constructing clinical questions and selecting outcome indicators, evidence retrieval, generation of recommendations, *etc*.

## Introduction

Headache is either a disease or a clinical symptom of multiple diseases.^[1]^ Acute primary headache causes pain in the organism, and long-term, persistent headache is a trigger and aura manifestation for many highly disabling diseases^[2,3]^ The current treatment for acute primary headache is primarily analgesia. As no clinical practice guideline exists specifically for the treatment of acute primary headache using Traditional Chinese Medicine (TCM), this guideline was developed to recommend TCM treatments for acute primary headache aimed to relieve pain and standardize clinical behaviors of physicians, and allied health professionals who treat or care patients with acute primary headache in outpatient/emergency/in-patient care. It aims to recommend TCM for the treatment of acute primary headache, and improve the management, clinical efficacy, and quality of life in patients with headache.

Syndrome differentiation and modification are critical in improving the efficacy of TCM in treating acute primary headache. However, such clinical evidence is difficult to obtain through randomized controlled trials (RCTs) or systematic reviews. Ancient literature is a treasure trove of TCM, an important basis for decision making in TCM clinical practice, and has a wide range of guidance for the treatment of acute primary headache using TCM. Therefore, the ancient TCM literature was also used as a major element of clinical evidence in the development of this guideline.^[4]^

This guideline focuses on acute primary headache and provides recommendations for their treatment using TCM, which can be used as a guide and reference for clinical practice by physicians in TCM internal medicine, neurology, and other related departments, and it will be revised according to clinical practice and advances in evidence-based medicine. The guideline

does not function as a medical code of conduct or a legal basis. Clinical practice based on this guideline may have different benefits for different patients and may produce different clinical outcomes.

## Methodology of Guideline Development

### Guideline registration

The guideline proposals were registered on the practice guideline registration for transparency (PREPARE) (guidelines-registry.org/), with registration number IPGRP-2021CN156.

### Guideline working group

The guideline working group was founded in June 2021, with four subgroups: guideline steering committee, guideline development group (GDG), guideline secretary group, and guideline review group. The working group members are multidisciplinary experts, not only involving clinicians and methodologists but also due consideration on the values and wishes of potential conflicting parties and patients.

### Guideline steering committee

The guideline steering committee comprises 10 to 15 experts. It has two chairpersons: professional and methodological chairpersons. All experts signed a conflict of interest statement, and at least one of the chairpersons did not have a conflict of interest related to this guideline. The main responsibilities of the guideline steering committee are as follows: (1) determine the topic and scope of the guideline; (2) approve the guideline proposals; (3) manage its conflict of interest statement; (4) oversee the guideline development process; (5) approve the guideline recommendation; and (6) approve the guideline publication.

***Guideline development group***

The GDG is composed of multidisciplinary clinical experts and methodologists. The main responsibilities of the GDG are as follows: (1) identify population, intervention, comparative measures, and outcome indicators and determine the scope of the guideline; (2) draft the guideline proposal; (3) collect and select clinical questions and outcome indicators, and construct clinical questions according to patient/population problem, intervention, comparison or control, and outcome (PICO) principles; (4) assess the importance of clinical questions and outcome indicators; (5) develop systematic reviews, evidence grading, and decision tables; (6) investigate patients’ wishes; (7) determine quality grading of evidence and reach consensus on recommendations; (8) write the full text of the guideline, complete the coordination of the external review of the guideline, and handle the external review comments; (9) document the whole process of guideline development; and (10) manage matters related to guideline development.

### Guideline external review group

The external review group contains relevant experts and stakeholders, and its main task is to review the developed guideline recommendations.

### Statement and management of conflict of interest

A conflict of interest statement form was made. All members of the guideline steering committee and guideline development group are required to fill in the form and agree to present the conflict of interest statement and the results of its handling as an annex to the guideline. For those who have a conflict of interest, the guideline steering committee decides whether or to what extent they would participate in the development of the guideline after discussion; those who do not have a conflict of interest could participate in the whole process.

### Purpose, target population, and users of the guideline

The purpose of this guideline is to recommend TCM treatment for acute primary headache, to guide clinicians and nursing staff to standardize the use of TCM for the treatment of acute primary headache with the main purpose of pain relief, and to improve the management of patients with headache, clinical efficacy, and the quality of life of patients. Users of the guideline include physicians and nursing staff of TCM internal medicine, neurology, and other related departments. The target population of the guideline is patients who were diagnosed with acute primary headache.

### Scope of the guideline

The clinical questions covered in this guideline relate to the TCM treatment of acute primary headache. The diagnosis of headache is based on the diagnostic criteria of headache disease in the Development of Internal Medicine of Traditional Chinese Medicine,^[5]^ which includes a class of diseases with headache as the main clinical feature caused by external factors and internal injury resulting in constriction or loss of nourishment of the veins and channels or by unfavorable clearing of the orifices. Western medicine diagnosis is based on the International Classification of Headache, Third Edition.^[6]^ Primary headache includes migraine, tension-type headache, trigeminal autonomic headache, and other primary headaches. This guideline does not cover secondary headaches, painful cranial neuropathy, other facial pain, or other types of headaches. This guideline applies to the pharmacological treatment of ongoing headaches and does not apply to the prevention of headaches during remission in patients with headache. Interventions include oral and topical TCM treatments.

### Construction of clinical questions

The working group investigates the importance of the collected and summarized PICO clinical questions and outcome indicators after reviewing relevant guidelines and surveying clinicians, multidisciplinary experts, and patients. The survey respondents are multidisciplinary experts and patients/guardians who rate the importance of clinical questions and outcome indicators. The importance of the outcome indicator is assessed through the method recommended by the GRADE working group.^[7]^ The selection of clinical problems will be completed through multiple rounds of questionnaires.

### Evidence retrieval

In this guideline, published relevant guidelines, systematic reviews, and related literature published domestically and internationally in the past 3 years are retrieved based on the questions covered. Evidence evaluation will be performed based on the results of systematic reviews if the evidence in existing guidelines is not sufficient to answer the clinical questions posed in this guideline; relevant literature will be retrieved by the guideline development group to produce systematic reviews if high-quality systematic reviews in the past 3 years are not retrieved. Evidence from ancient sources lacking clinical studies is retrieved by an antiquarian literature agency based on the Classification Standard of Evidence in Ancient Books of TCM,^[8]^ and a retrieval report will be produced. The National Library of Medicine (PubMed), the Cochrane Library, and the Cochrane CENTRAL database (The Cochrane Central Register of Controlled Trials), Guideline International Network (GIN), National Guideline Clearinghouse (NGC), National Institute for Health and Clinical Excellence (NICE), Scottish Intercollegiate Guide Network (Scottish Intercollegiate Guidelines Network, SIGN), World Health Organization (WHO) database, Wanfang, China National Knowledge Infrastructure (CNKI), China Biomedical Literature Database (SinoMed), and Chinese Sci-tech Journal Database (VIP), Baidu Xueshu (https://xueshu.baidu.com/), Yimaitong (https://www.medlive.cn/), and Zhonghua Yidian (TCM Dictionary) version 5.0 will be searched. Specific retrieval strategies are developed based on the characteristics of different databases using a combination of subject terms and free words. The development of retrieval strategies and the literature retrieval are conducted under the guidance of a literature retrieval specialist. No language limitation is applied. In addition, references from included studies are traced to gain access to relevant literature. Information such as relevant retrieval strategies and results are reported in a formal guideline document.

### Evidence evaluation

The methodological quality of the included guidelines is evaluated using the GRADE approach.^[9]^ The evidence quality will be categorized as “high”, “moderate”, “low”, or “very low”. The evaluation process is completed independently by at least three people, and the coefficient of internal consistency among the evaluation team members is calculated. The methodological quality of the included systematic reviews re-review, systematic reviews, meta-analyses, and network meta-analyses were evaluated using the AMSTAR scale.^[10]^ The evaluation of the ancient evidence is completed by the guideline development group according to the TCM ancient evidence standards. The evaluation process is completed independently by two members, and if there is disagreement, it is discussed together, or a third party is consulted to resolve it. If no systematic reviews related to the clinical questions is retrieved, GDG will make a new round of systematic reviews to answer the clinical questions.

### Recommendations

The guideline development group develops a “Problem-Recommendations-Evidence” decision form, sets the expert opinions as “Agree, Disagree, and Uncertain,” and submits it to the expert steering committee. The expert steering committee will reach consensus on the recommendations by telephone, email, or face-to-face method.

### External review

The guideline recommendations will be submitted to the external review team for feedback, and the feedback will be reviewed by the guideline steering committee to determine and publish the final version of the recommendations of the guideline.

### Implementation and dissemination of the guideline

After the guideline is reviewed and revised by the expert steering committee based on the external review, the full text of the guideline will be published in relevant journals, popular science journals, and other relevant mass media. Dissemination will be performed by holding relevant conferences and training courses.

### Update of the guideline

The guideline is planned to be updated every 5 years.

## Conclusion

The development group of TCM Guidelines for Acute Primary Headache will raise clinical questions through the real needs of clinicians in the practice of TCM for acute primary headache. The group raises clinical questions, performs evidence retrieval, quality evaluation, and recommendations, and formulates high-quality and practical clinical practice guidelines, considering the opinions of relevant experts, the wishes of patients with headache, and economic factors, to guide the clinical TCM treatment of acute primary headache and improve clinical outcomes of patients.
